# Design and in vitro, in vivo evaluation of antioxidant bioadhesive gels for burn treatment

**DOI:** 10.55730/1300-0152.2613

**Published:** 2022-01-17

**Authors:** Göksel GÖKÇE, Sinem Yaprak KARAVANA, Alper BAĞRIYANIK, Çetin PEKÇETİN, Evren ALGIN YAPAR, Gülşen AYBAR TURAL, Evren Homan GÖKÇE

**Affiliations:** 1 Department of Pharmacology, Faculty of Pharmacy, Ege University, Bornova, İzmir, Turkey; 2 Department of Pharmaceutical Technology, Faculty of Pharmacy, Ege University, Bornova, İzmir, Turkey; 3 Department of Histology-Embryology, Faculty of Medicine, Dokuz Eylül University, İzmir, Turkey; 4 Department of Pharmaceutical Technology, Faculty of Pharmacy, Cumhuriyet University, Sivas, Turkey; 5 İzmir Biomedicine and Genome Center (IBG), İzmir, Turkey

**Keywords:** Taurine, antioxidant, bio-adhesive gel, burn wound model

## Abstract

Burn wounds are frequently encountered health problems, which need a new treatment approach especially in terms of good patient compliance. Availability of use of antioxidant agents and bio-adhesive gels in tissue healing can be an alternative as a new approach for wound healing. Antioxidant taurine containing bio-adhesive gels were prepared by using carbopol (CP) 940 and 934. Rheological and texture analyses were carried out on bio-adhesive gels for in vitro characterization. Wound model on Wistar rats was used to evaluate the in vivo evaluation of gels. Rheological and texture analyses showed that a carbopol bioadhesive gel has acceptable topically use dosage characteristics and in combination with Taurine it presented a successful wound healing effect via antioxidant parameters. In conclusion, bio-adhesive CP 940 (2%) gel containing 50 mM taurine could be promising in the treatment of burns by balancing oxidative stress.

## 1. Introduction

Acute wounds (surgical wounds, burns, cuts, and various traumatic wounds) and chronic wounds (diabetic wound and venous wound) are frequently encountered health problems. Although the annual incidence of burns is not known exactly, it is estimated to be 200,000 patients in Turkey and about 400,000 hospital admissions per year in the United States (Martin, 1997; Salinas et al., 2008; White and Renz, 2008; Fitzmaurice et al., 2011). The priorities of the treatment of burns focus on to stabilize the patient, prevent the infection, and optimize the recovery (D’Avignon et al., 2010).

Healing of all kinds of wounds is similar in terms of being a dynamic process with overlapping phases. In the initial inflammatory phase, neutrophils and monocytes immigrate to the site of injury via localized vasodilation and fluid extravasation, thereby starting an immune response that is later sustained by the recruitment of macrophages by chemokines (Rowan et al., 2015).

The inflammatory phase does not only prevent infection during healing, but it is also necessary to degrade the necrotic tissue and initiate signalization for healing of the wound (D’Avignon et al., 2010). In this dynamic process, the role of antioxidants has long been investigated. Current thinking supports a correct balance between oxidative and antioxidative forces (Tiwari, 2012). The free radicals combat invading microorganisms and aid in cellular signaling. Hence, the tissue concentrations of these free radicals should be precisely controlled to avoid the cellular damage that occurs with excess oxidative stress (Poljsak, 2013). Studies have shown that antioxidant agents are effective in tissue healing. It is thought that epithelialization will accelerate, especially if the oxidative stress is balanced (Dunnill et al., 2017). Thus, in this study taurine was used as an antioxidant to test this approach and for this aim, it is incorporated in a bio-adhesive gel. Taurine (2-aminoethanesulfonic acid), an antioxidant agent, is an organic acid, an essential component of bile acids, and it is widely synthesized in many tissues in mammals. Taurine is a derivative of cysteine, a sulfur-containing amino acid, and is the only sulfonic acid known to form in the body. Taurine has physiological importance in osmoregulation, bile acid conjugation, modulation of central nervous system functions, cell proliferation, and prevention of oxidative damage in tissues (Portugal et al., 2007; Çetinkalp et al., 2021).

Bio-adhesive systems have advantageous properties compared to conventional medicinal forms in terms of localization in the target area for a long time, thus increasing bioavailability, modifying absorption properties, reducing the frequency of dosing, and providing treatment compliance (Wang et al., 1996).

Bio-adhesive hydrogels are three-dimensional, hydrophilic, polymeric network-shaped structures that can absorb large amounts of water and adhere very well to the desired area. They are suitable carrier systems for water-soluble active substances and as the water evaporates, a cooling effect occurs; this feature can also be used in the treatment (Zatz et al., 1996).

Carbomers (carbopol polymers: CP) are acrylic acid polymers that are used as rheology modifiers, tablet binders, suspension stabilizers, extended-release polymers, mucoadhesive aids, and bioavailability enhancers. Carbopol polymers are well known for their versatility and ease of use as pharmaceutical excipients. Products within the carbopol polymer family are chemically similar in that they are all high molecular weight, crosslinked polyacrylic acid polymers. CP 934 and CP 940 are both homopolymers of acrylic acid crosslinked with allyl sucrose or allyl pentaerythritol. CP934 and CP940 perform different viscosity at the same conditions (%0.5 wt% concentration at pH = 7.5, 30500–39400cP and 40000–60000cP, respectively (Gaikwad et al., 2012). These two types were selected to evaluate the differences between the mechanical properties of gels.

In this study, bio-adhesive gel formulations prepared with two different types of carbomers containing taurine were designed and in vitro characterization was performed via rheological and mechanical tests. Moreover, in vivo healing performance of gels was investigated in rats with second-degree burns and antioxidant parameters were evaluated.

## 2. Materials and methods

### 2.1. Materials

Taurine (Sigma Aldrich, Turkey), carbopol 940 (CP 940) and carbopol 934 (CP 934) (Lubrizol, Germany), triethanolamine (Merck, Turkey), hematoxylene/eosin were of pharmaceutical grade. Antioxidant parameter kits GSH-420 (GSH), Catalase-520, GPx-340 (GPx), LPO-586 (LPO) were purchased from Bioxytech, USA. Other chemicals were of analytical grade.

### 2.2. Preparation of bio-adhesive gels

CP 940 and CP 934 were preferred as the most widely used polymers in the preparation of bio-adhesive gels. CP 934 or CP 940 was slowly added to distilled water at a concentration of 1% or 2% (w/w) at 600 rpm in small portions and mixed with a magnetic stirrer for 3–5 h at room conditions. The resulting homogeneous mixture was left for one night to complete the swelling of polymer. The pH value of the gel formulation was adjusted to pH = 7 with triethanolamine to complete the gelation process.

Taurine incorporated gels were prepared by solubilizing taurine in water at a concentration of 50 mM and by adding polymers at a concentration of 1% or 2% (w/w) as previously described. The formulations were mixed until homogeneous and were centrifuged at 3000 rpm for 15 min to remove air bubbles. The samples were kept in the refrigerator for one day before the necessary analyzes for their characterization were conducted (Tan et al., 2000; Değim et al., 2002). Formulations of bio-adhesive gels are given in Table 1.

### 2.3. Characterization of bio-adhesive gel formulations

#### 2.3.1. Rheological analyzes of bio-adhesive gel formulations

**Figure 1:**
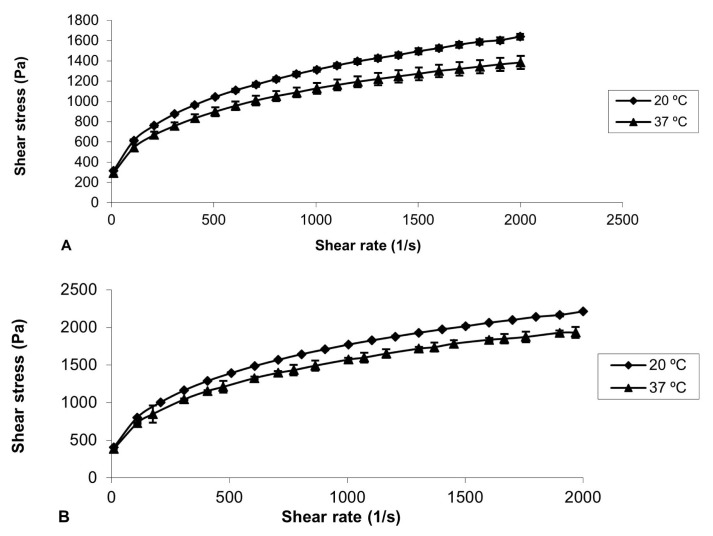
Flow reograms of bio-adhesive gel formulations at 20 °C ve 37 °C. A) J1-1b (1% CP 940), B) J1-2b (2% CP 940).

**Figure 2:**
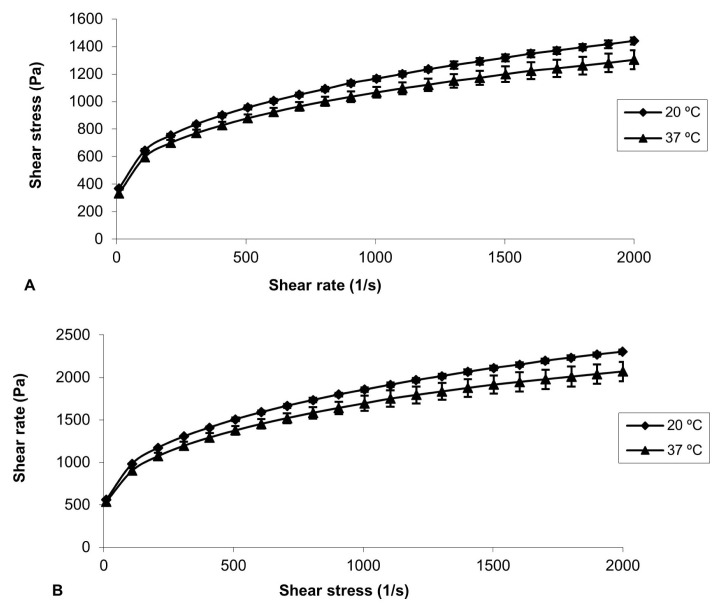
Flow reograms of bio-adhesive gel formulations at 20 °C and 37 °C. A) J2-1b (1% CP 934), B) J2-2b (2% CP 934).

**Figure 3:**
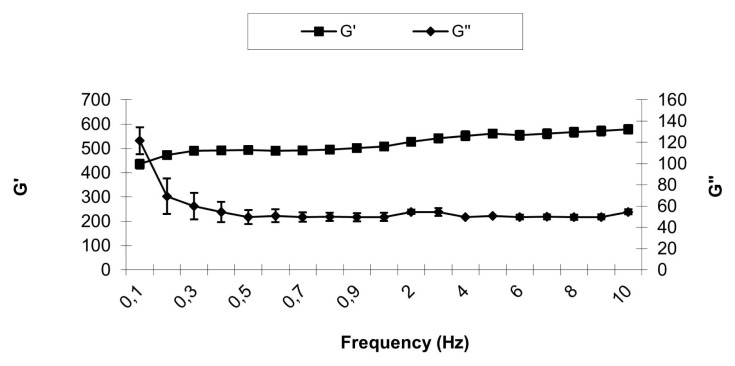
Frequency-dependent changes in viscoelastic properties of J1-2b gel prepared with 2% CP940 and 50 mM taurine at 37°C. G′, storage modulus; G″, loss modulus.

The rheological analyzes of the formulations were carried out at 20 ± 0.1°C and 37 ± 0.1°C using the AR 2000 stress/ rate-controlled rheometer (HAAKE MARS Modular Advance Rheometer System) with a 40 mm diameter parallel steel probe in flow mode, keeping the 0.3 mm gap constant. During the run, the samples were carefully applied to the plate of the rheometer and kept for 5 min before starting the measurement to ensure that the flow of the formulation was set to a minimum. Oscillation analysis was performed for each formulation at 20 ± 0.1°C and 37 ± 0.1°C, in the linear viscoelastic region of that formulation, where the stress was directly proportional to the strain and the storage modulus remained constant (Figure 1 and 2). In the frequency scanning analysis, the frequency was changed in the range of 0.1–10.0 Hz, while the stress was kept constant. At the end of this analysis, elastic modulus (G′), viscous modulus (G″), dynamic viscosity (ŋ ′) and phase angle (tan δ) were calculated with the Rheology Advantage Software program provided by Instruments (New Castle, DE, USA) (Figure 3). This study was repeated at least 3 times for all gel formulations.

#### 2.3.2. Texture profile analysis (TPA)

The mechanical properties of the gels were determined using the TA-XT Plus Texture analyzer (TA-XT Plus, Stable Micro System, UK) equipped with a 5 kg load cell in texture profile analysis mode (Karavana et al., 2009). The following parameters were used for the test. The analytical probe was twice inserted into formulations to a defined depth (8 mm) and at a defined rate (1.5 mm/s), allowing a delay period (15 s) between the end of the first and beginning of the second compression. Using the obtained power-time curve, the hardness, adhesiveness, cohesiveness, elasticity, and compressibility of the gels were calculated (Tables 2 and 3).

### 2.4. In vitro scratch assay

The scratch assay was performed to examine the effects of gels on wound healing in vitro. Human keratinocytes were seeded in sterile cell culture plates with 24 wells and incubated in standard cell culture conditions, at 37 °C, 5% CO_2,_ and 95% humidified atmosphere.

50 mM taurine incorporating 2% CP 940 gels (J1-2) in comparison to control and blank gel (J1-2b) were sterilized at 121°C for 15 min in an autoclave and diluted to 0.1g/mL in DMEM high glucose. 10% fetal bovine serum, 1% sodium pyruvate, and 1% penicillin-streptomycin were added to the samples.

**Figure 4:**
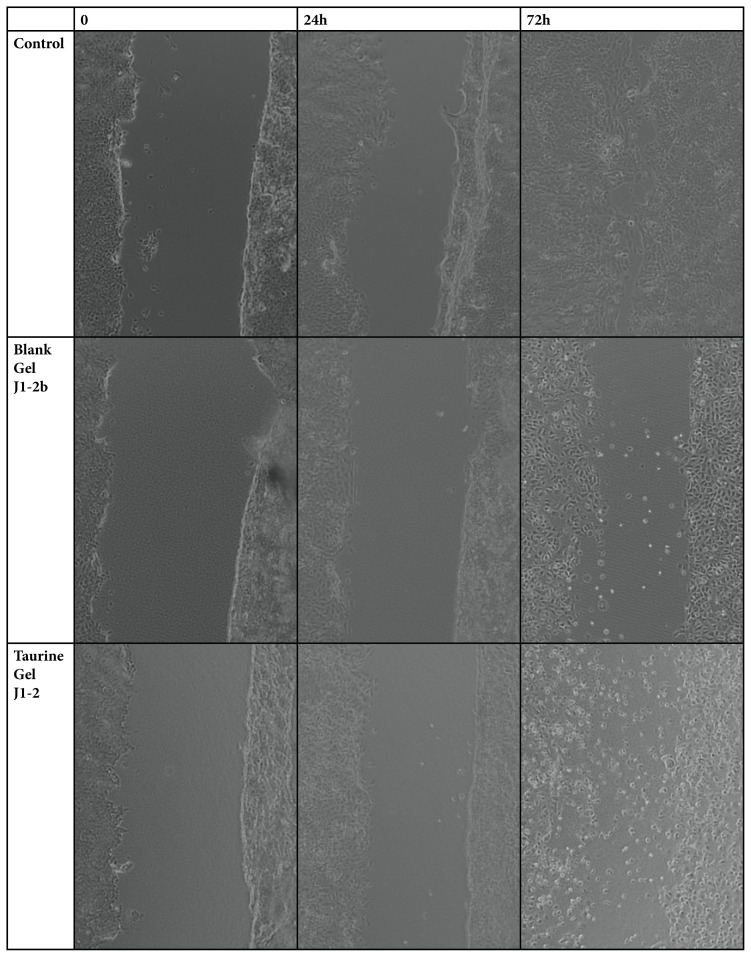
Wound closure extent and cell migration zone images of HACAT cells after 24 h and 72 h of incubation with gels with a magnitude of 10x. The control group is untreated cells.

After reaching the 100 % confluence, a wound (scratch) was created in a cell monolayer, in the middle of each well. The cells were then washed with phosphate buffer saline, and gels were added. The cells were incubated with samples and the effects on wound closure, and cell migration were monitored for 72 h. Microscopic analysis of wound closure was performed on the inverted light microscope (Axio, Zeiss, Germany) (Figure 4). The extent of wound closure - determined by calculating the ratio between the surface area of the wound three days after incubation with samples and the area of the initial wound, just before the addition of samples.

### 2.5. In vivo studies in rat wound model

In vivo studies were performed according to Declaration of Helsinki for the use of animals in research and approved by the Local Animal Ethical Committee (Approval No: 2011–16). Wistar rats having 6 rats in each group were divided into 4 groups as follows: a) Control group (C1), b) Burn group (C2), c) Empty gel group, and d) Treatment group (J1-2). In the control group (C1), wound model was not formed on rats. The burn group (C2) consisted of animals with burn models without any treatment.

The rats were anesthetized using ketamine/xylazine (60/8 mg/kg, IM) before conducting the experiments. The backs of the anesthetized rats were shaved, and cylindrical molds with a base area of 3.14 cm^2^ with open upper and lower parts were placed on the backs of the animals, and 95 °C water was put into the mold and contacted on the backs of the animals for 15 s. The wounds were closed with sterile sponges (Abdullahi et al., 2014). Once a day, 1g of bio-adhesive gel containing 50 mM Taurine (J1-2) or 1g of bio-adhesive gel (J1-2b) without taurine was applied onto the burned area for 7 days.

**Figure 5:**
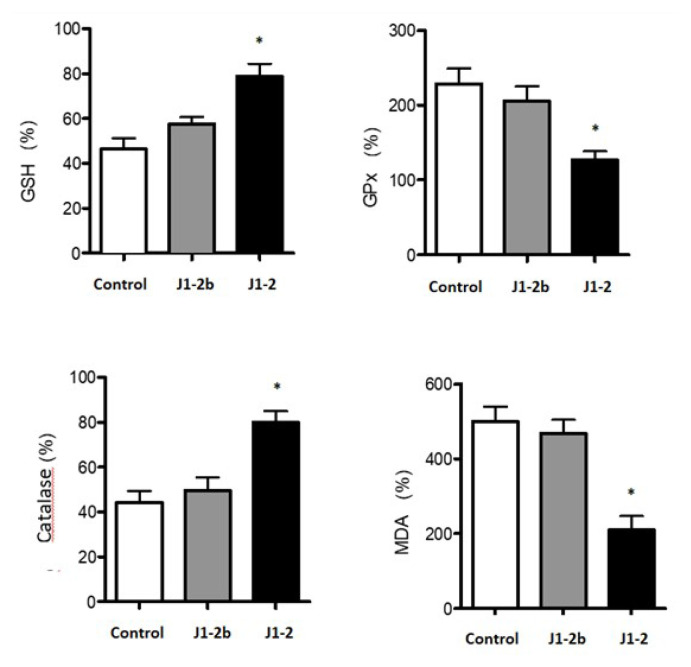
Antioxidant parameters of Taurine incorporated into 2% CP940 prepared gels (J1-2b) in comparison to control and blank gel (J1-2b). * p < 0.05 vs. control and blank gel. GSH: Glutathione; GPx: Glutathione peroxidase; MDA: Malondialdehyde; Control: the burn model group; J1-2b: 2% CP 940 blank gels; J1-2: 50 mM Taurine incorporating 2% CP 940 gels.

Biochemical parameters were determined spectrophotometrically in homogenates obtained from skin tissue (Figure 5). The skin tissue in the burned area was dissected from the surrounding connective tissue and frozen in liquid nitrogen. Tissues were kept at −86 °C until further process. Tissues were homogenized using a sonicator (Bandelin Sonoplus, UW 2070, Germany) for 2 min after the addition of the lysis buffer solution included in the commercial kits to be used to measure biochemical parameters. The amount of protein in the supernatant was separated by centrifugation at 14000 g for 20 min and it was measured by the Bradford method (Bradford, 1976).

Antioxidant parameters are shown in Figure 5 for the following tests.

#### 2.5.1. Glutathione (GSH) determination

Commercially marketed kits were used for GSH measurements (Bioxytech, GSH-420, Oxis Research, USA). The method is based on thion formation, which is a chromophore group (Okatani et al., 2000). The absorbance measured at 420 nm is directly proportional to the GSH concentration. The method has three stages: 1) Ensuring the conversion of GSSG into GSH by adding a reducing agent, tris-2-carboxyethyl phosphine, to the sample in the buffer solution (potassium phosphate, diethylenetriamine pentaacetic acid, lubrol, pH: 7.8), 2) conversion of thiols in the sample to thioethers by adding chromogen (4-chloro-1-methyl-7-trifluoromethylquinolinium methyl sulfate), 3) increasing the pH above 13 by adding base solution (sodium hydroxide) and the GSH-thioether group forms the thion.

#### 2.5.2. Catalase determination

Commercially marketed kits were used to measure catalase levels. (Bioxytech, Catalase-520, Oxis Research, USA). The conversion of hydrogen peroxide to water and molecular oxygen is proportional to the concentration of catalase. The basis of the method is based on stopping the reaction with sodium azide after a 1-min incubation of the sample with a hydrogen peroxide solution of known concentration (Mattson et al., 2002). After these processes, the hydrogen peroxide remaining in the reaction mixture was reacted with 4-aminophenazone and 3,5-dichloro-2-hydroxybenzenesulfonic acid under horseradish peroxidase (HRP) catalysis. The amount of catalase in the sample was calculated from the concentrations of the formed quinonimine dye corresponding to the absorbance levels at 520 nm.

#### 2.5.3. Glutathione peroxidase (GPx) determination

Commercially available kits were used to measure glutathione peroxidase (GPx) levels (Bioxytech GPx-340, Oxis Research, USA). The method is based on the indirect calculation of cellular GPx activity (Hoyos et al 2000). Oxidized glutathione (GSSG) formed by GP catalysis is converted to its reduced form, GSH, in the presence of glutathione reductase (GR). The conversion of GSSG to GSH is accompanied by the oxidation of NADPH to NADP+. Since this oxidation reaction causes a decrease in absorbance at 340 nm, it allows the GPx activity to be monitored. The molar depletion coefficient for NADPH is 6220 M^−1^ cm^−1^ at 340 nm. In our study, the enzyme reaction was initiated with ter-butyl hydroperoxide by adding lysates obtained from tissue samples to a solution containing GSH, GR, and NADPH, and the absorbance at 340 nm was recorded. The decrease in absorbance is directly proportional to the GPx activity.

#### 2.5.4. Lipid peroxidase (MDA) determination

Lipid peroxidation is a widely used marker to determine the level of oxidative stress in cells and tissues. Commercially available kits were used to determine the level of lipid peroxidation (Bioxytech LPO-586, Oxis Research, USA). Lipid peroxides are molecules that are structurally unstable and tend to degrade into a range of compounds containing reactive carbonyl groups. Peroxides of polyunsaturated fatty acids form malondialdehyde (MDA) and 4-hydroxyalkenales (HAE) after said conversion. The method allows the determination of MDA alone (using hydrochloric acid) or in combination with HAE (using methane sulfonic acid) (Mattson et al., 2002). The measurement was carried out by determining the absorbance of the samples at 586 nm.

#### 2.5.5. Histomorphological studies

The samples taken from the skin tissue obtained from the burned area, and other control groups were fixed in 10% neutral buffered formalin for 3 days and routine tissue follow-up was started. After washing under running water for 1 night to remove the fixative, it was passed through a series of ethyl alcohol that increased by 70%, 80%, and 96%, respectively, for 20 min at 60 °C. Then, it was dehydrated in acetone for 20 min in an oven at 60 °C. It was kept in xylol for 30 min in an oven at 60°C for transparency purposes. It was immersed in paraffin for 1 hour in an oven at 60 °C and embedded in paraffin blocks. Sections of 5 μm were taken using a rotary microtome. Sections of each subject were stained with hematoxyline-eosin to evaluate the general histomorphological features of the tissue.

**Figure 6:**
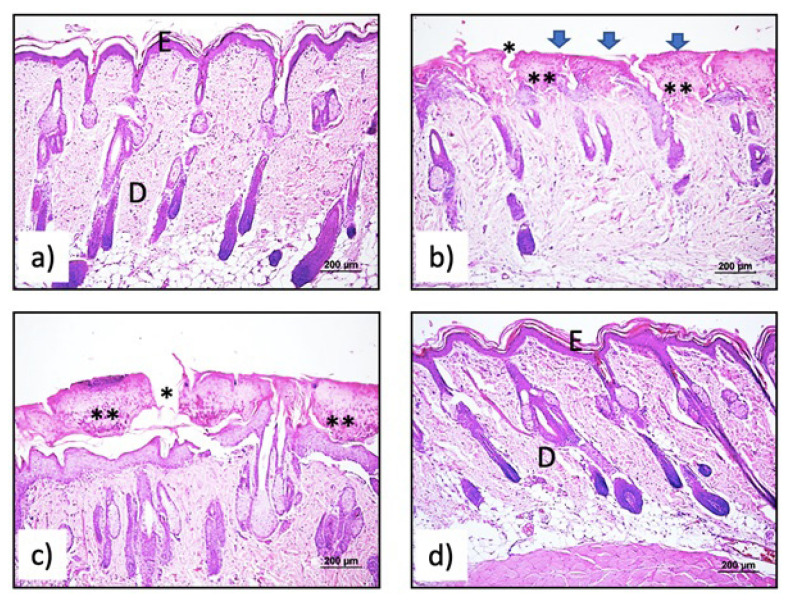
a) Normal skin tissue of the control group (C1), E: Epidermis, D: Dermis. H&E x10. b) In the burn group (C2), thinning of the epidermis, subepidermal separations (*), with fibrin deposition (arrow heads) on the epidermis and polymorphonuclear leukocyte infiltration crust formation (**) H&E x10. c) Subepidermal separations (*) in the skin tissue belonging to the empty gel group and polymorphonuclear leukocyte infiltration (**) H&E x10 d) Skin tissue of the treatment group (J1-2) H&E x10.

The sections taken were left in an oven at 60 °C for 2 h for deparaffinization. Then, they were exposed to three different xylenes, the first of which was 20 min (in the oven) and the other two were 10 min each. Then, for the rehydration process, it was passed through to exchange absolute alcohol and alcohol series decreasing from 96% to 70%. After rinsing the sections with distilled water, they were stained with Hematoxylin for 10 min. After staining, sections washed in running water for 10 min to remove excess dye from the tissue were stained with Eosin dye for 2 min. After staining, the sections passed through 70%, 80%, 96%, and 2 series of absolute alcohol series, respectively, were kept in three changes of xylene for 20 min for transparency, and then closed with entellan. The preparations were evaluated histologically. Computerized video camera-based image analysis method was used to analyze the images obtained from the sections (UTHSC Image software). After the staining was completed, the sections were transferred to the computer with the help of a high-resolution camera connected to the light microscope [Aver TV Studio Video Capture (Version 4.21.0.0 (Software) Aver Media Technologies, Inc.)]. All sections were photographed digitally (Figure 6).

The healing in the burned area was evaluated with a scoring developed by Hosseini et al. (2011). Re-epithelialization, granulation, inflammatory cell formation, and angiogenesis were scored between 0 and 4. The value 0 represented the worst, and 4 was for the best parameter score (Table 4). They were examined in 4 groups: C1 (control tissue), C2 (burned tissue, no treatment applied), J1-2b (Blank gel), and J1-2 (Taurine gel).

### 2.6. Statistical analyzes

All statistical analyzes were evaluated using the Tukey test method, following one-way analysis of variance (ANOVA). Significant difference level was taken as 95%. (p < 0.05).

## 3. Results and discussion

In our study, bioadhesive gels were prepared with CP 940 and CP 934 at two different concentrations, 1% and 2%, to investigate the effect of concentrations. It is very important to carry out rheological analyses of the prepared gels to examine both the flow properties and the oscillation properties. The data obtained from these studies give information about the stability of the preparations during storage and temperature changes. In our study, these analyses were performed at 20 ± 0.1°C (storage temperature) and 37 ± 0.1°C (application site temperature) (Perioli et al., 2008). In the flow study, changes in shear stresses were detected despite the increasing shear rate of the gels. At the end of the flow studies with Carbopol gels, pseudoplastic flow was observed (Figure 1 and 2). This result is compatible with the literature (Deasy et al., 1991; Zatz and Kushla 1996).

Semi-solid preparations applied must have certain mechanical properties for the patient to benefit as much as possible. The easy removal of the gel from the primary packaging and the ability of the product to remain in the desired area for a long time without disintegration are important parameters in the design of adhesive gels. The basic physical mechanism of bio-adhesion depends on the flexibility of the polymer chain.

It was determined that the interaction between shear rate and shear stress increased depending on the increase in molecular weight. In this study, the same interaction was observed due to the increase in the amount of carbopol. There are records in the literature that flow studies cause fragmentation of the system due to the applied rotational motion, thus making the system more fluid (Jones et al., 2003).

Flexible polymer chains enter between the polymer and the mucus, forming a stronger adhesive bond. The elasticity value of bio-adhesive gel formulations complements the time-dependent direction of the gel’s structural recovery after deformation (Jones et al., 1997; Jones et al., 2003).

In the study of Bonacucina et al., (2006), it is stated that the adhesive value of gels varies depending on the viscosity of the bio-adhesive polymer, and gels prepared with polymers with high viscosity have higher adhesive values. The results of our study were in accordance with these results.

TPA studies were carried out to evaluate the mechanical properties of the formulations such as being able to come out of the package, spreading and persistence at the application site, the hardness, adhesiveness, cohesiveness, and compressibility of carbopol gels. The adhesive value of a bio-adhesive formulation should be high, as it demonstrates its ability to adhere to tissue. During TPA analysis, adhesiveness refers to the force required to release the probe from the bio-adhesive gel (Jones et al., 2003).

The increase in the numerical value of the elasticity obtained during TPA analysis indicates that the elasticity of the gel decreases as the polymer concentration decreases. It is desired that bio-adhesive gel formulations have high elasticity. A similar relationship was seen in our formulations depending on the concentration. It has been found that the presence of active ingredients reduces its compressibility, being softer and less adhesive than blank gels. CP 940 performed higher compressibility, adhesiveness, and hardness at the same concentration range as CP934. It was concluded that the viscosity of the polymer might have this effect on the mechanical properties. As the result of the studies, it was determined that J1-2b was the most ideal gel formulation and since the highest adhesion was obtained with J1-2b (Table 2 and Table 3), and 50 mM Taurine was incorporated into this optimized formulation. Taurine is a sulfur-containing amino acid present in high concentrations in mammalian plasma and cells, plays an important role in several essential biological processes such as development of the central nervous system and the retina, calcium modulation, membrane stabilization, reproduction, and immunity (Schuller-Levis & Park 2003). In fact, taurine is the single most abundant amino acid in leukocytes at a concentration of 20–50 mM, thus 50mM of taurine was added into gels.

In vitro scratch assay: The scratch assay was used to evaluate the wound healing potential of gels. One of the main reasons for the prevalence of the scratch assay is its simple protocol. In this assay, a scratch is introduced into the confluent cell monolayer by using a sharp object such as a pipette tip (Stamm et al., 2016).

Hydrogels of pectin, gum arabic, calcium chloride dihydrate with and without fibroblast growth factor, were evaluated with scratch assay at 12 h. There was an obvious wound closure for the bioinspired hydrogels at 12 h (Zhang et al., 2018).

Studying dermal fibroblast migration may help to target therapies for improved wound healing. The scratch assay showed that the C940 gel alone was not effective; however, the addition of Taurine increased the closure of the wound. Similar to the results of this study, Taurine gel was shown to have the ability to improve the cell repair process and tissue regeneration (Ashkani-Esfahani et al., 2014; Niu et al., 2018). It was observed from the images that, during 72h of exposure, there was a statistically significant difference in the wound healing effects between the blank and taurine incorporated gels. However, for the first 24 h, no wound healing could be seen in both groups, only the traces of cell migration could be observed for Taurine incorporated gels. The difference between examined samples in wound closure extent and cell migration zones at 24h and 72 h can be seen in Figure 4.

In vivo studies: In wound healing studies with experimental animals, it has been observed that the gel containing taurine provided a very dramatic improvement compared to the group treated with the blank gel without taurine and the untreated group. After utilizing the burn model, C940 gel containing taurine was applied to the wound area every day for 1 week and healing was observed. No excessive crusting was observed in the taurine-administered group, however wound healing could be observed with crusting in the Taurine-free gel group and control group from the 3rd day. This situation was thought to be caused by the osmoregulatory effect of taurine. It is thought that while it replaces the water loss in the cell in a balanced way, it may also have developed a protective effect against the naturally occurring radical oxygen species in the wound area. The improvement seen in the blank gel group was far behind the untreated group. This is thought to be due to the triethanolamine used for neutralization. In previous trolox equivalent antioxidant capacity studies containing co-enzyme Q10, it was observed that the antioxidant activity of co-enzyme Q10 was suppressed by triethanolamine (Korkmaz et al., 2013). In order to observe the results of this effect on the tissue, the tissues were taken and examined in terms of antioxidant parameters.

GSH, GPx, catalase, and MDA ratios were calculated in skin tissue homogenates, and values were expressed in % compared to control. The burning significantly decreased GSH and catalase levels and increased MDA and GPx levels. Blank gel did not differ significantly for any of the parameters compared to the burned control group (p > 0.05). The Taurine-containing gel (J1-2), on the other hand, brought all parameters closer to the control group consisting of normal rat skin samples (Figure 5; p < 0.05 vs. control and blank gel). At the end of one week, improvement was observed in all animals treated with Taurine-containing gel. At this point, tissues were taken to determine formulation effects, and these differences were determined by histology studies (Figure 6).

Images of normal skin tissue were observed in the control group (Figure 6a). In the burn wound group (C2), thinning of the epidermis, subepidermal separations, fibrin deposition on the epidermis, and polymorphonuclear leukocyte infiltration, crust formation, and necrotic areas were observed (Figure 6b). While thickening of the epidermis was observed in the blank gel group (J1-2b), polymorphonuclear leukocyte infiltration and dermal-epidermal separations continued (Figure 6c). The images of normal skin tissue were comparable with taurine treatment group. Reepitelization, was significantly pronounced for taurine incorporating gel (J1-2) treated group.

**Figure 7:**
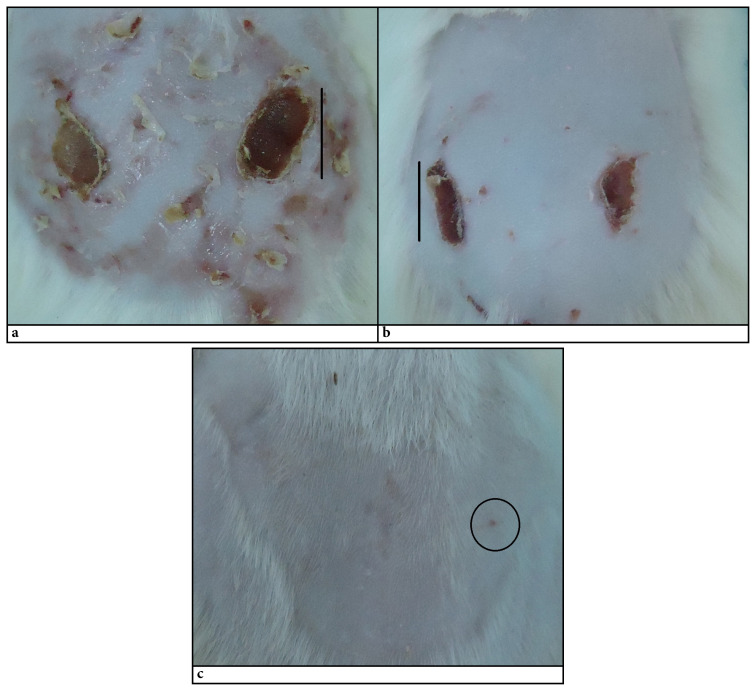
The skin images of the albino Wistar rats at the end of the 7th day of in vivo experiments. a) Burned but not treated group, C2-control b) C940 blank gel group (J1-2b), c) Taurine gel group (J1-2). The bars are 1 cm long. The circle is showing the place of the wound.

The effect of Taurine could be obviously seen in Figure 7, indicating the wound healing without crust formation. The scoring was conducted according to method developed by Hosseini et al. (2011). Reepithelialization, granulation, inflammatory cell formation, and angiogenesis were scored. The normal skin tissue was evaluated as 4 for all parameters. Blank (J1-2b) group had slightly higher results than burn group (C2) for all parameters, but none of them exceed 2 (Table 5). Although the healing was more pronounced for J1-2b group, overall scoring profile was similar to C2. The effect of taurine was obvious when compared to treatment groups(C1). Reepithelialization and granulation were over 3, and inflammatory cell and angiogenesis parameters were almost double the value of blank and burn groups.

## 4. Conclusion

Antioxidant agents are effective in the healing of burns; thus, bio-adhesive hydrogels of taurine were prepared with different polymers, and optimum formulation was determined by in vitro characterization studies. Taurine-free gel and taurine incorporated gel were applied on Wistar rats employed for the wound model and analyzed by scoring the healing process. The tissues were homogenized, and the level of oxidative stress was determined via GSH, catalase, GPx, and MDA levels. In addition, tissues obtained were histologically evaluated in terms of indicators of wound healing. As a result, it can be stated that bio-adhesive CP 940 (2%) gel containing 50 mM taurine could be promising in the treatment of burns with suitable mechanical properties without crust formation. Further clinical studies and in vivo efficacy studies should be planned.

## Figures and Tables

**Table 1:** Formulation codes and contents of bioadhesive gels prepared with CP 940 and CP 934.

Code	CP 940 (%)	CP 934 (%)	Taurine (mM)
J1-1b	1	---	---
J1-1	1	---	50
J1-2b	2	---	---
J1-2	2	---	50
J2-1b	---	1	---
J2-1	---	1	50
J2-2b	---	2	---
J2-2	---	2	50

**Table 2:** TPA results of bioadhesive gel formulations at 20 °C.

Code	Hardness (N) ± SS	Adhesiveness (N.mm) ± SS	Cohesiveness ± SS	Compressibility (N.mm) ± SS	Elasticity ± SS
J1-1b	0.471 ± 0.034	0.885 ± 0.083	0.957 ± 0.030	1.084 ± 0.145	0.658 ± 0.058
J1-2b	0.610 ± 0.022	1.476 ± 0.105	0.937 ± 0.019	1.681 ± 0.096	0.693 ± 0.044
J2-1b	0.363 ± 0.009	0.801 ± 0.077	0.962 ± 0.027	0.970 ± 0.055	0.806 ± 0.044
J2-2b	0.395 ± 0.025	0.781 ± 0.087	0.957 ± 0.006	0.910 ± 0.107	0.654 ± 0.039

**Table 3:** TPA results of bioadhesive gel formulations at 37 °C.

Code	Hardness (N) ± SS	Adhesiveness (N.mm) ± SS	Cohesiveness ± SS	Compressibility (N.mm) ± SS	Elasticity ± SS
J1-1b	0.454 ± 0.029	0.866 ± 0.235	0.933 ± 0.043	1.004 ± 0.195	0.698 ± 0.076
J1-2b	0.593 ± 0.033	1.466 ± 0.182	0.920 ± 0.052	1.350 ± 0.118	0.832 ± 0.128
J2-1b	0.349 ± 0.009	0.783 ± 0.084	0.927 ± 0.028	0.913 ± 0.097	0.829 ± 0.042
J2-2b	0.385 ± 0.015	0.667 ± 0.086	0.934 ± 0.013	0.808 ± 0.066	0.674 ± 0.081

**Table 4:** Scoring parameters conducted for in vivo studies.

	Reepithelialization	Granulation	Inflammatory cell/histological field	Angiogenesis
**0**	Absence of proliferation	Immature tissue	13–15 cells	Presence of hemorrhage and edema
**1**	Poor epidermal organization	Thin immature tissue	10–13 cells	1–2 vessels per site, edema, hemorrhage, and congestion
**2**	Incomplete epidermal organization	Moderate remodeling	7–10 cells	3–4 vessels per site, moderate edema, congestion
**3**	Moderate proliferation	Thick granulation layer	4–7 cells	5–6 vessel per site slight edema, congestion
**4**	Complete epidermal remodeling	Complete tissue organization	1–4 cells	More than 7 vessels per site, vertically disposed toward epithelial surface.

**Table 5:** Findings of the scoring study. C1: control tissue, C2: burned tissue, no treatment applied, J1-2b: Blank gel, and J1-2: Taurine gel.

Group (n:4)	Reepithelialization	Granulation	Inflammatory cell	Angiogenesis
**C1**	4	4	4	4
**C2**	0.5	1.4	0.9	1
**J1-2b**	0.8	1.6	1.3	1.35
**J1-2**	3.45	3.15	2.7*	2.5*

* p < 0.05 J1-2 vs C1
